# Supplementary figures and images for: Analysis of carbon emission performance and regional differences in China’s eight economic regions: Based on the super-efficiency SBM model and the Theil index
Source: PLoS One. 2021 May 5;16(5):e0250994. doi: 10.1371/journal.pone.0250994 (PMC8099138; doi:10.1371/journal.pone.0250994)

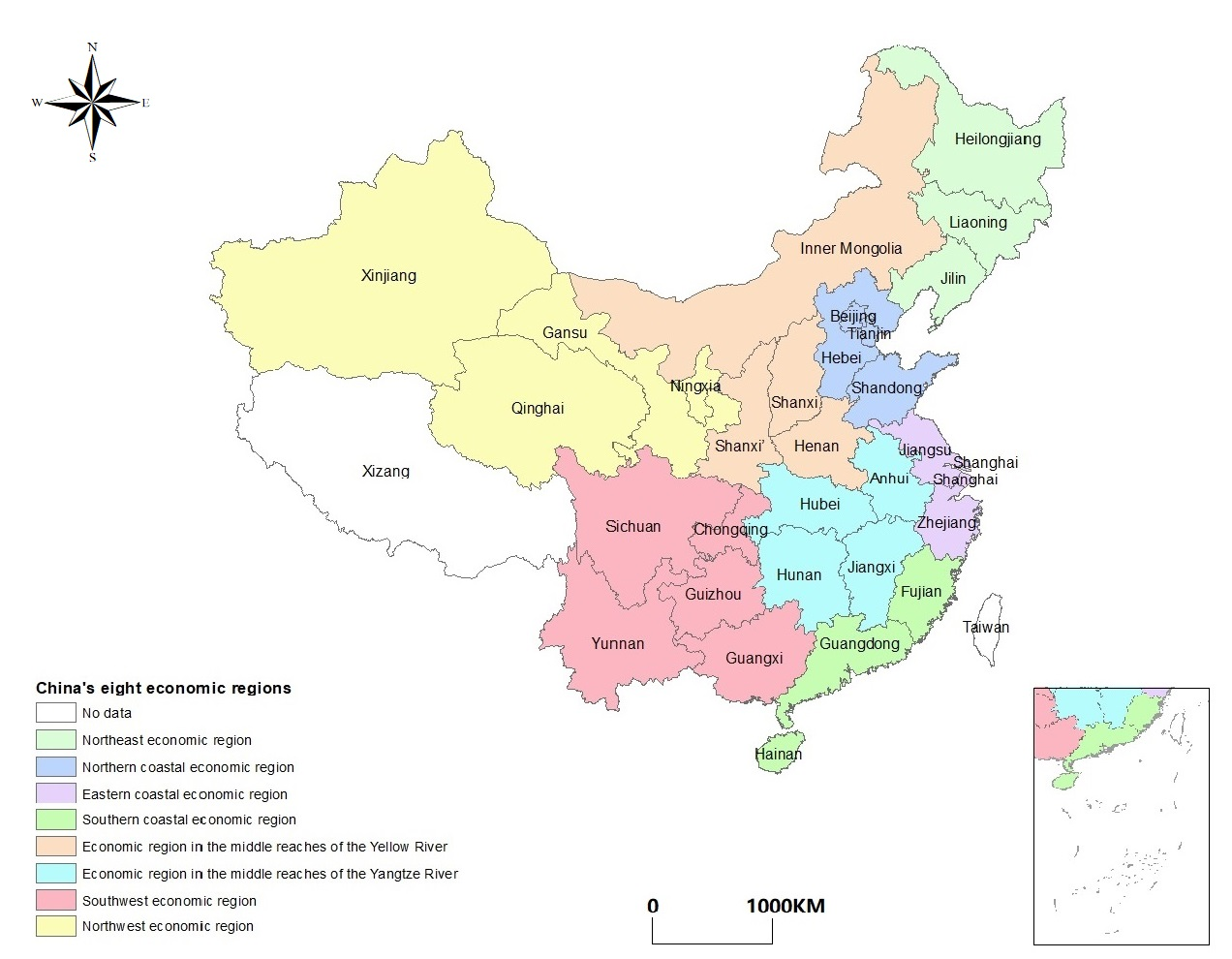

Supplement: S1 Fig — (TIF) [file pone.0250994.s001.tif]

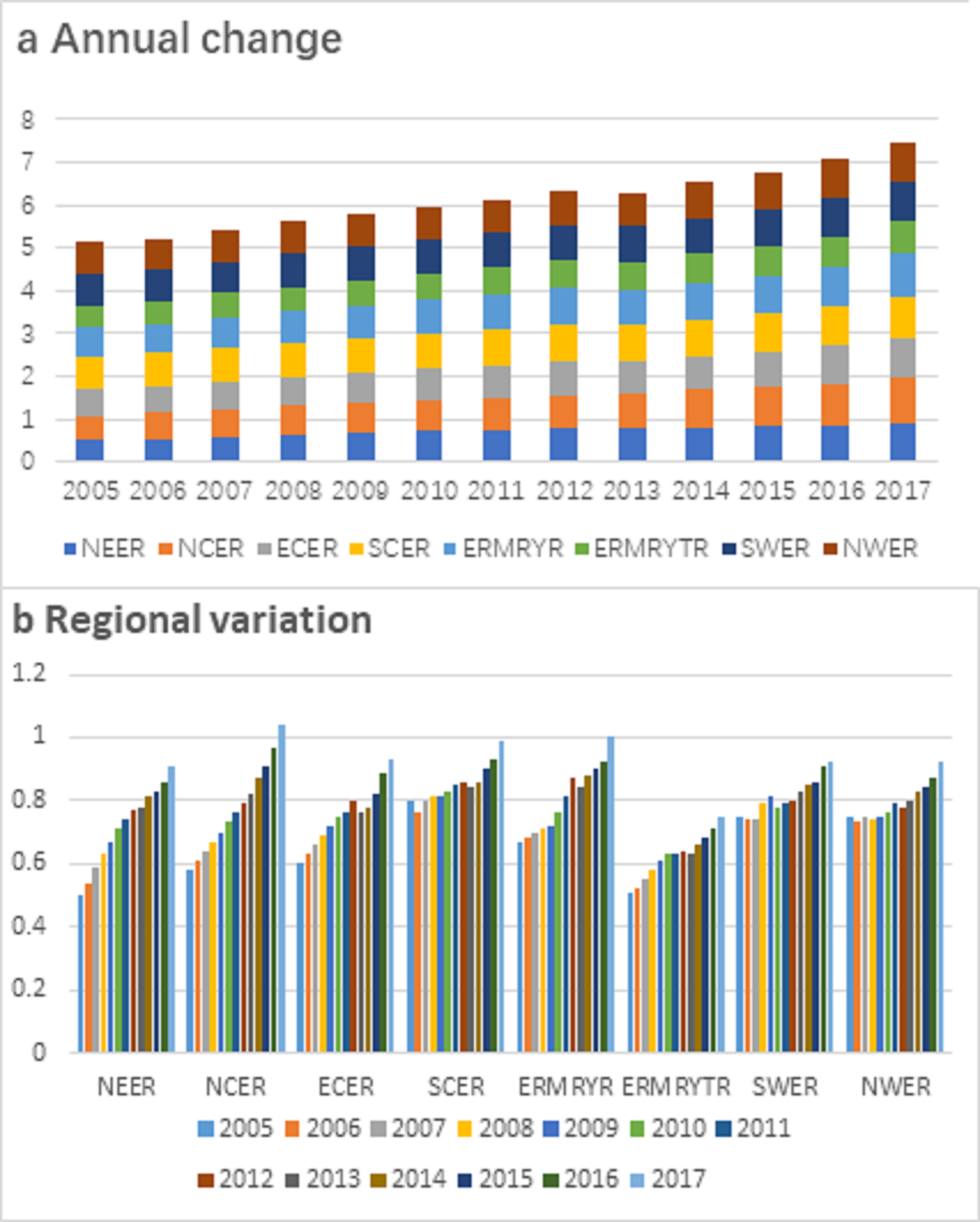

Supplement: S2 Fig — (TIF) [file pone.0250994.s002.tif]

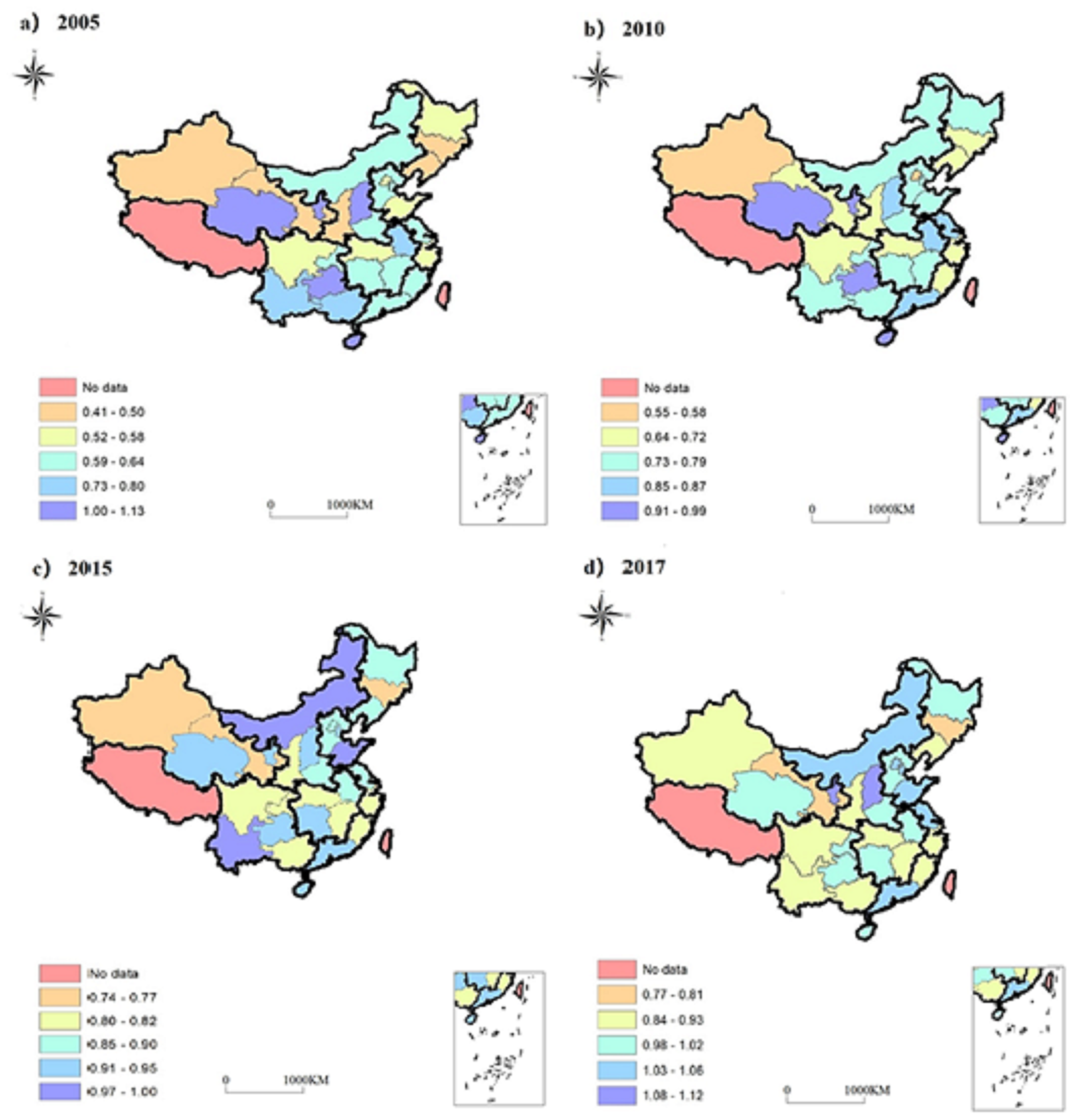

Supplement: S3 Fig — (TIF) [file pone.0250994.s003.tif]

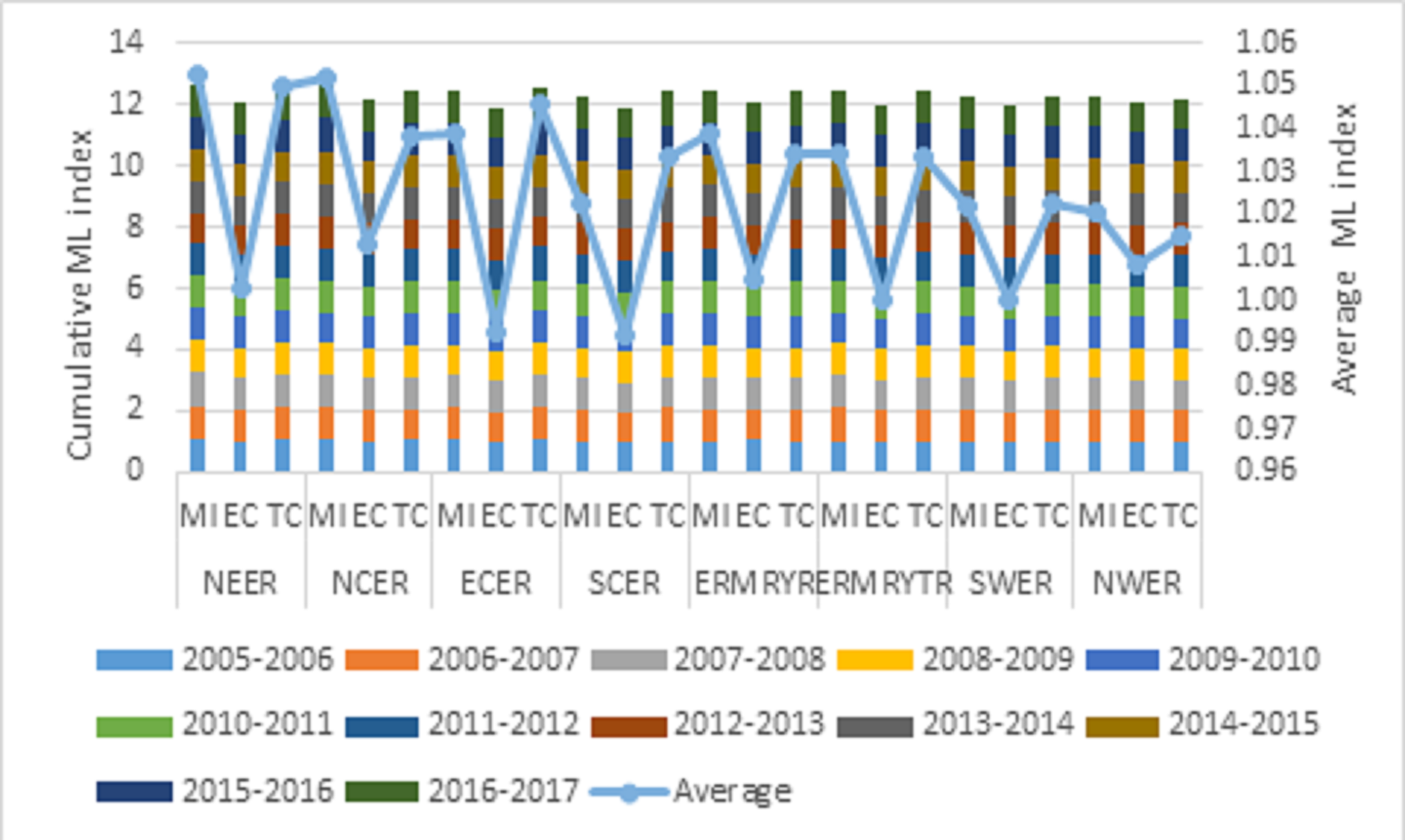

Supplement: S4 Fig — (TIF) [file pone.0250994.s004.tif]

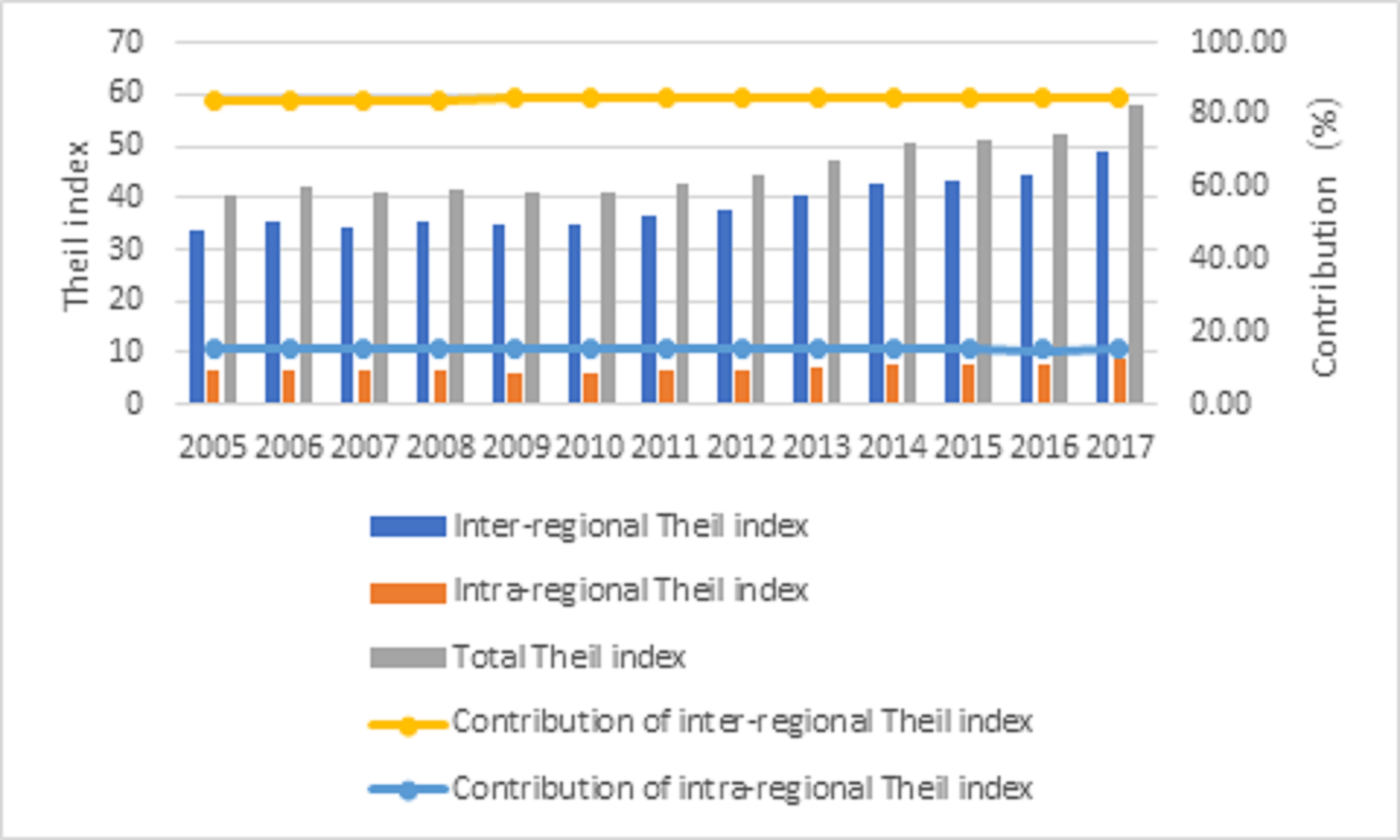

Supplement: S5 Fig — (TIF) [file pone.0250994.s005.tif]

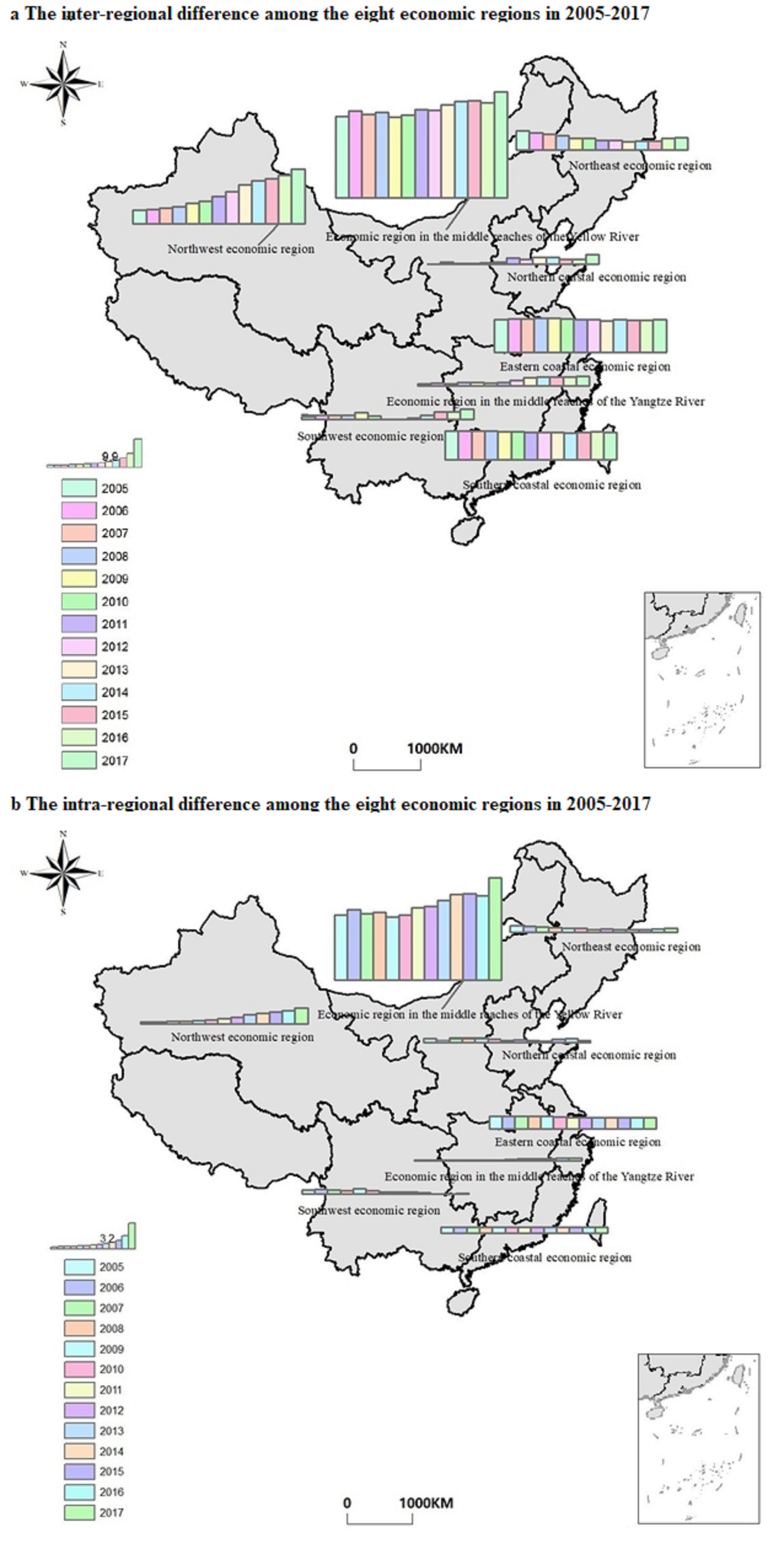

Supplement: S6 Fig — (TIF) [file pone.0250994.s006.tif]

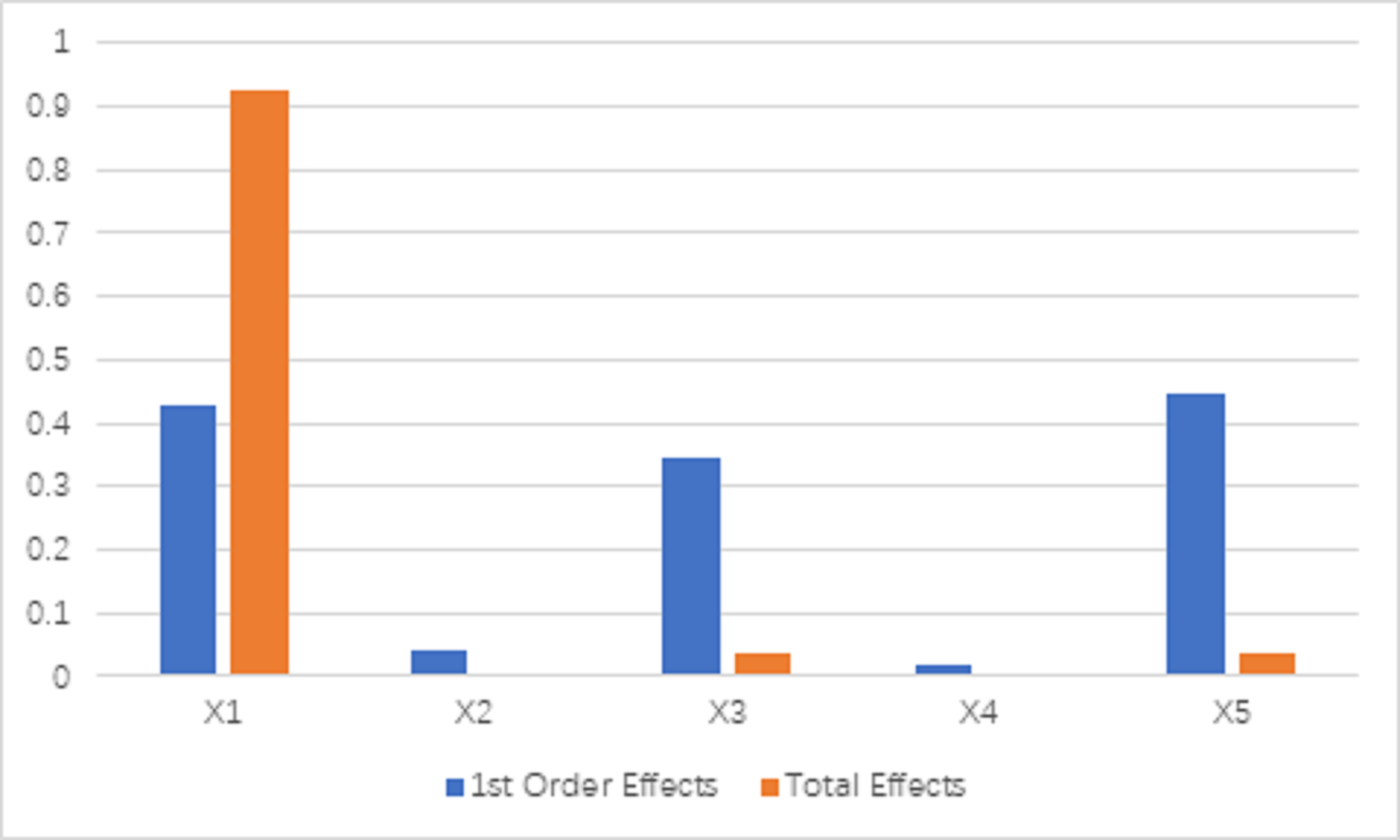

Supplement: S7 Fig — (TIF) [file pone.0250994.s007.tif]
